# The invasive GAS puzzle in Italy: genomic insights from a hospital cohort in a fragmented surveillance landscape

**DOI:** 10.3389/fcimb.2025.1684665

**Published:** 2026-01-07

**Authors:** Roberto Rosato, Giulia Santarelli, Barbara Fiori, Francesca Romana Monzo, Giordana Cafaro, Tiziana D’Inzeo, Marilena La Sorda, Roberta Creti, Brunella Posteraro, Maurizio Sanguinetti, Flavio De Maio

**Affiliations:** 1 Dipartimento di Scienze Biotecnologiche di Base, Cliniche Intensivologiche e Perioperatorie, Università Cattolica del Sacro Cuore, Rome, Italy; 2 Dipartimento di Scienze di Laboratorio ed Ematologiche, Fondazione Policlinico Universitario A. Gemelli IRCCS, Rome, Italy; 3 Microbiota Analysis & Microbial WGS Research Core Facility, GSTeP, Fondazione Policlinico Universitario A. Gemelli IRCCS, Rome, Italy; 4 Dipartimento di Malattie Infettive, Istituto Superiore di Sanità, Rome, Italy; 5 Unità Operativa “Medicina di Precisione in Microbiologia Clinica”, Direzione Scientifica, Fondazione Policlinico Universitario A. Gemelli IRCCS, Rome, Italy

**Keywords:** iGAS, M1UK, *Streptococcus pyogenes*, surveillance program, WGS

## Abstract

*Streptococcus pyogenes* (Group A *Streptococcus*, GAS) is a re-emerging human pathogen responsible for a wide spectrum of diseases, from mild pharyngitis to life-threatening invasive infections that have risen globally in recent years. We performed a retrospective genomic and epidemiological analysis of 61 invasive Group A *Streptococcus* (iGAS) isolates collected from 2016 to 2024 at a major tertiary Italian hospital. Whole-genome sequencing, emm typing, phylogenetic reconstruction, and mutational profiling were performed to assess clonal diversity, virulence determinants, antimicrobial resistance (AMR) genes, and mobile genetic elements. Our data revealed high genetic diversity, with multiple emm types identified, including emm1.0, emm28.0, emm89.0, emm22.21, and emm44.0 being the most prevalent. Notably, four isolates belonged to the hypervirulent M1UK sub-lineage, which has circulated in Italy since 2018 without a marked post-pandemic resurgence. Genome-wide analysis identified multiple genomic alterations. Regulatory gene mutations were widespread, particularly in *rexB* (97%), *covS* (82%), and *rexA* (80%) suggesting a complex modulation of virulence pathways. AMR genes were sporadic and largely absent in emm1.0 and M1UK strains, showing 12 or 26 core SNPs, reinforcing the hypothesis that virulence, rather than resistance underpins their clinical relevance. Our findings depict a genetically diverse and evolving *S. pyogenes* population in Italy, dominated by a few high-risk lineages. The persistence of M1UK strains without explosive spread underscores the influence of local epidemiological contexts and surveillance limitations. This study emphasizes the urgent need for a national genomic surveillance network to enable timely detection of emerging clones and guide public health responses.

## Introduction

GAS infections vary in severity, encompassing asymptomatic and mild cases (such as impetigo, pharyngitis, and scarlet fever) and more severe illnesses, referred to invasive GAS (iGAS), (such as pneumonia, streptococcal toxic shock syndrome, and necrotizing fasciitis) and autoimmune disorders (Brouwer et al., 2023). Notably, iGAS infections account for roughly 1.8 million cases worldwide, with a mortality rate as high as 20%, affecting both young and elderly populations (C.f.D.C.a. Prevention, 2025). Unfortunately, these infections are often preceded by non-specific symptoms such as fever and pharyngodinia (Wessels, 2022). Following a significant increase in cases beginning in late 2022 and extending into 2023 across several European countries and beyond, *Streptococcus pyogenes* (*S. pyogenes*) has become a pathogen of escalating global concern in the post-pandemic years. Notably, an increase in iGAS infections has been reported, which disproportionately impacted pediatric populations (Johannesen et al., 2023). The trend was seemingly caused by a combination of factors, including the “immune debt” resulting from non-pharmaceutical interventions during the COVID-19 pandemic, shifts in respiratory viral circulation, and the spread of highly virulent emm types of *S. pyogenes* (Cohen et al., 2023; Munro and House, 2024; Rumke et al., 2024; De Arcos-Jimenez et al., 2025; de Crombrugghe et al., 2025).

Although previous epidemiological data exist, significant gaps exist in the early detection and tracking of circulating strains, especially in countries without established surveillance frameworks (Massese et al., 2024). In Italy, the lack of a nationwide program to monitor GAS infections makes it difficult to evaluate past trends and the genetic variation of circulating strains, particularly before the pandemic. Several countries in Europe, including the UK, France, and the Netherlands, have implemented nationwide obligatory reporting systems for iGAS, facilitating timely epidemiological notifications and genomic surveillance (De Maio et al., 2024). These discrepancies hamper both intra- and inter-country comparability and undermine efforts toward coordinated public health responses. Against this background, a growing understanding is emerging of whole-genome sequencing as a universal tool for determining the genetic profile of *S. pyogenes*, differentiating between persistent, recurrent, or emerging strains, and pinpointing genomic markers of heightened virulence or antibiotic resistance (De Maio et al., 2024).

Few sequencing data for *S. pyogenes* are currently available in Italy, including both invasive and respiratory strains (De Maio et al., 2025b). This absence represents a significant gap in the genomic surveillance of this microorganism in comparison with other pathogens (https://www.iss.it/registri-e-sorveglianze). One contributing factor is that, at present, only scarlet fever is a notifiable disease in the country, while iGAS are not subject to mandatory reporting so that epidemiological data on *S. pyogenes* are limited and fragmented.

This study sought to genetically define the range of invasive *S. pyogenes* strains isolated at a major Italian hospital over almost eight years (2016-2023) and place our results within Italy’s overall epidemiological patterns. We aimed to capture the local spread of *S. pyogenes* and the potential public health consequences by combining clinical, microbiological, and genomic data. Our research highlights the pressing requirement for a unified, country-wide surveillance system and epidemiology studies that utilize genetic data to facilitate the implementation of preventative measures in tracking and containing this re-emerging infectious agent.

## Results

### Epidemiological characterization of iGAS strains isolated in the period 2016-2024

A total of 61 *S. pyogenes* isolates associated with invasive infections were collected between 2016 and 2024. Most strains were obtained from blood cultures of patients aged 1 to 89 years, with a nearly equal sex distribution, while no iGAS were isolated from other sterile anatomical sites. Of note, only two pediatric cases were recorded (**Supplementary Table 1**). Most samples originated from the Emergency Department (65.6%), followed by Obstetrics (4.9%), Internal Medicine (3.3%), and Geriatrics (3.3%).

**Figure 1:**
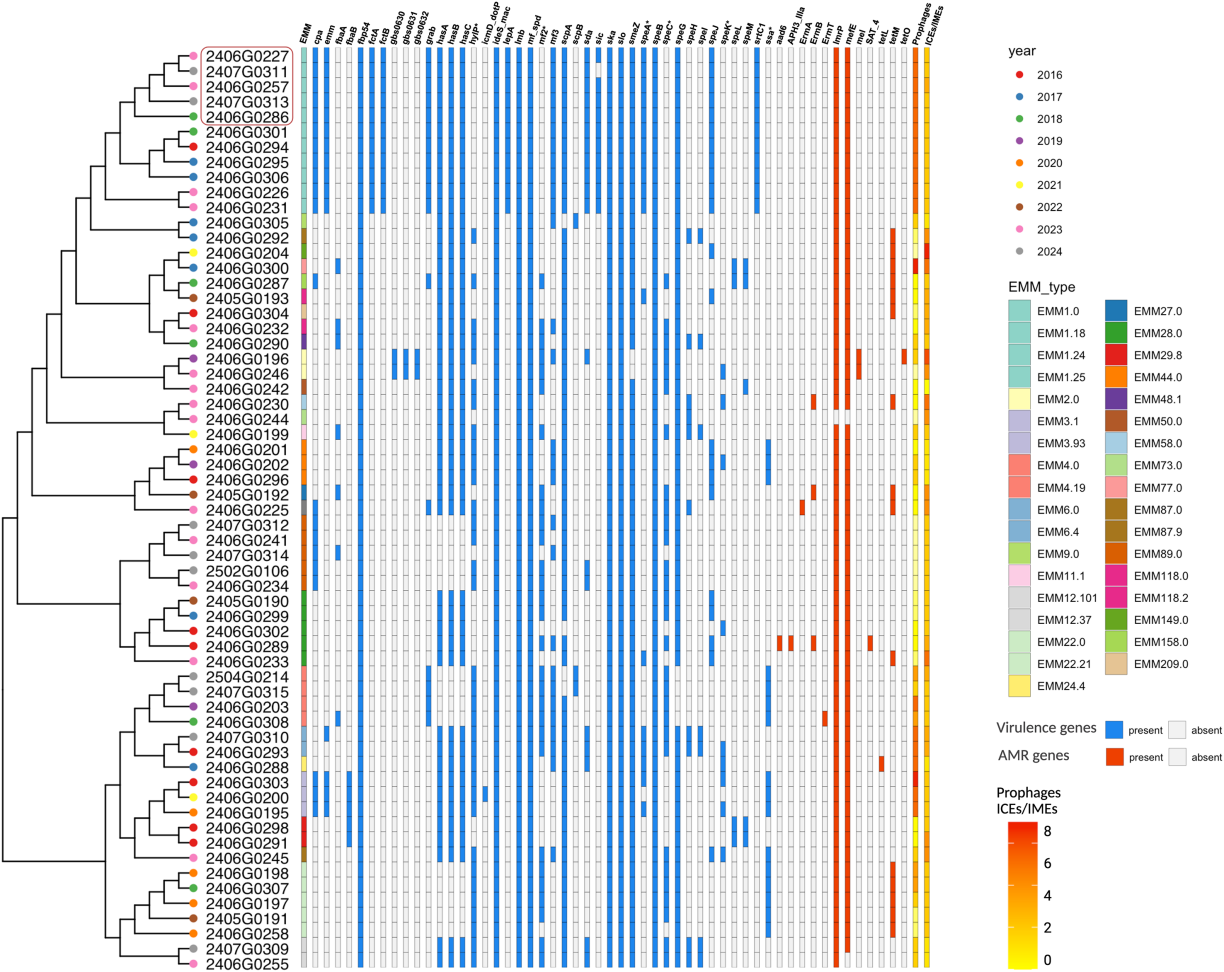
Phylogenetic and genomic integrative characterization of the invasive GAS isolates in our center. A maximum-likelihood phylogenetic tree was constructed from whole-genome sequencing data of invasive *Streptococcus pyogenes* isolates collected between 2016 and 2024. Each isolate is labelled with a unique code and colored according to the year of isolation. Each isolate’s EMM type is further represented. The heatmap adjacent to the phylogeny shows the presence or absence of virulence genes (blue) and antimicrobial resistance (AMR) genes (red). The gradient yellow-orange-red column on the far right accounts the number of prophages and integrative conjugative elements (ICEs) or integrative mobilizable elements (IMEs) detected in each genome. Red squares indicate M1UK isolates.

Whole-genome sequencing (WGS) and a single nucleotide polymorphism (SNP)-based neighbor-joining phylogeny revealed substantial genetic diversity among the isolates (**Figure 1**). Multiple emm types and their counterpart sequence types (STs) were identified, with emm1.0, emm28.0, emm89.0, emm22.21, and emm44.0 being the most prevalent. Notably, four emm1.0 and one emm1.24 isolates showed genetic signatures consistent with the hypervirulent M1UK lineage, characterized by 26 and 12 lineage-defining SNPs, respectively (**Supplementary Table 1**) (Li et al., 2023). These M1UK strains were detected starting in 2018, without evidence of a post-pandemic upsurge. Detailed metadata, including accession numbers, are provided in **Supplementary Data 1**.

Further analysis of genome-wide variants in emm1.0 isolates identified a broad range of mutational events: 91,600 SNPs, 6,559 multi-nucleotide variants (MNVs), 2,584 insertions, 2,373 deletions, and 741 complex replacements (**Supplementary Figure 1**; **Supplementary Data 2**). Single nucleotide variants (SNVs) and multi-nucleotide variants (MNVs) were the most common mutation types and were observed across all frequency ranges, including high-frequency bins (>50%). In contrast, insertions, deletions, and replacement events were predominantly found in low-frequency bins (<20%). SNVs showed a relatively uniform distribution, with representation even among the most frequent variants (>80%), indicating that some point mutations may be fixed or under positive selection in the population. Additionally, most mutations were sporadic and heterogeneously distributed rather than strain-specific, implying a highly diverse *S. pyogenes* population with a relatively conserved core gene (**Supplementary Data 2**). MNVs followed a similar but slightly more constrained trend. Conversely, insertions and deletions exhibited a strong skew toward low-frequency occurrence (<5%), suggesting that these types of mutations are more likely deleterious or subject to negative selection. Replacement mutations, which likely represent more disruptive changes, were also clustered in rare variant categories.

Although M1UK isolates phylogenetically clustered in a distinct branch, their virulence did not appear to correlate with the presence of specific virulence genes, prophages, or mobile genetic elements, suggesting that transcriptional regulatory mechanisms may underlie their enhanced pathogenicity ^12^.

**Figure 2:**
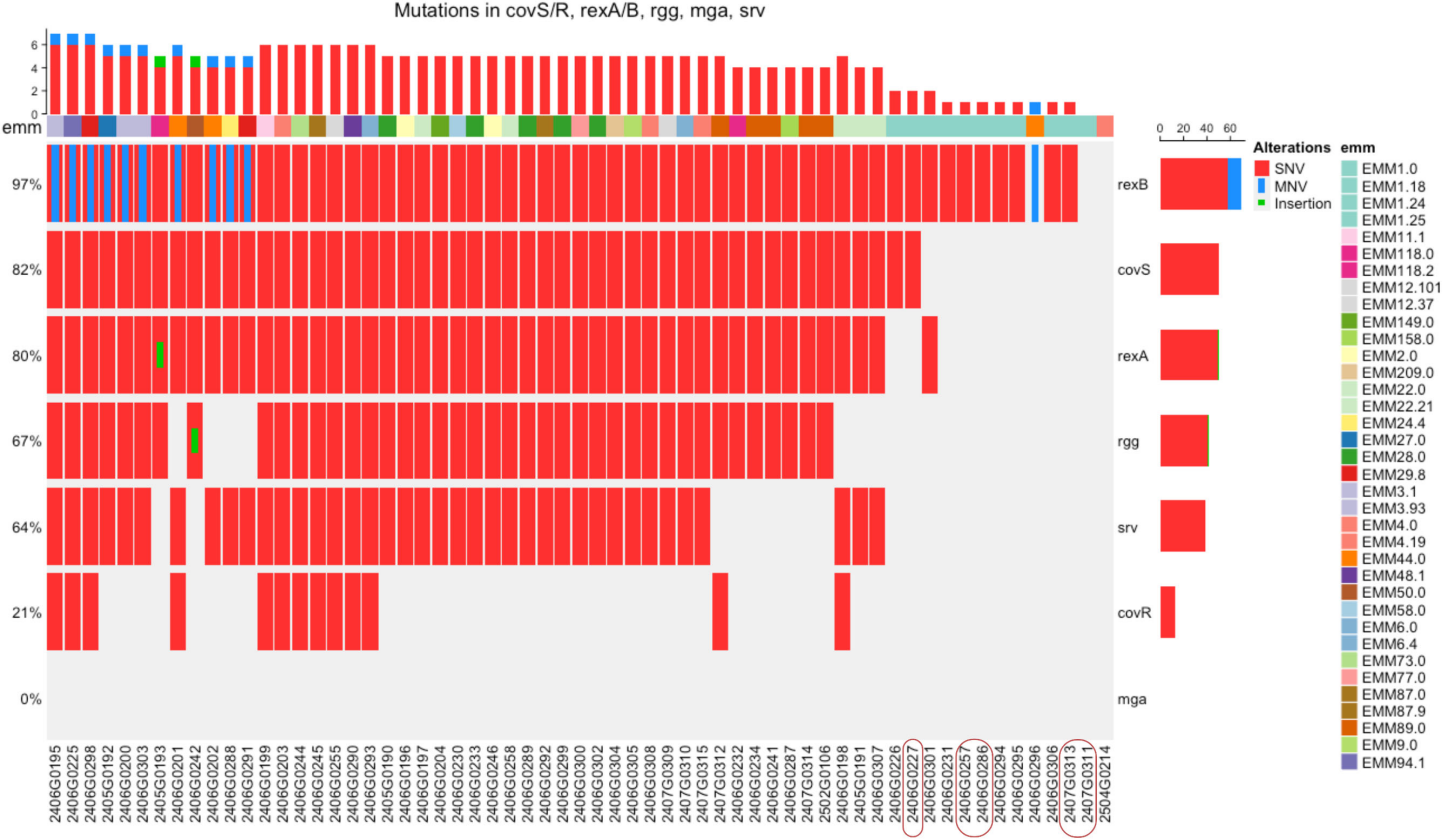
Distribution of mutations in regulatory genes across *Streptococcus pyogenes* clinical isolates grouped by *emm* type. The heatmap highlights the presence and distribution of genetic alterations in key virulence regulatory genes of *S. pyogenes*, specifically: control of virulence sensor kinase (*covS*), control of virulence response regulator (*covR*), repressor expression protein A (*rexA*), repressor expression protein B (*rexB*), regulator gene of glucose metabolism (*rgg*), streptococcal regulator of virulence (*srv*), and multiple gene regulator of virulence (*mga*). Each vertical column represents a clinical isolate, while each horizontal row corresponds to one of the regulatory genes analyzed. The presence of mutations is indicated by colored bars: red indicates single nucleotide variants (SNVs), blue indicates multi-nucleotide variants (MNVs) and green indicates insertions. The top bar plot shows the number and type of mutations per isolate, while the right-side bar plot summarizes the proportion of isolates harboring mutations in each gene. The horizontal annotation bar at the top categorizes each isolate by its EMM type. Red squares indicate M1UK isolates.

We analyzed the presence of mutations in seven key regulatory genes involved in *S. pyogenes* virulence modulation: control of virulence sensor kinase *(covS*), control of virulence response regulator (*covR*), repressor expression protein A (*rexA*), repressor expression protein B (*rexB*), regulator gene of glucose metabolism (*rgg*), streptococcal regulator of virulence (*srv*), and multiple gene regulator of virulence (*mga*) across a panel of clinical isolates grouped by emm type (**Figure 2**).

Comprehensive variant and annotation data for these regulatory genes are available in **Supplementary Data 3**.

Interestingly, a heterogeneous distribution of mutations was detected among the isolates. Indeed, most strains harbor mutations in at least one of the regulatory genes, with a clear predominance of SNVs compared to MNVs and insertions that appeared less common. Among the analyzed genes, *rexB* exhibited the highest mutation frequency, with alterations observed in over 97% of isolates. This was followed by *covS* (~82%) and *rexA* (~80%). Mutations in *rgg* and *srv* were detected in ~67% and ~64% of isolates, respectively. In contrast, *mga* and particularly *covR* showed none or lower mutation frequencies suggesting a crucial role for streptococcal viability. Of note, stratification by emm type highlighted that emm type 1.0 had the lowest density of mutations across multiple regulatory loci except for *rexB*, suggesting the potential association between the lack of mutations in regulatory genes with their virulence and fitness.

Among the emm1.0 isolates, including M1UK, a consistent virulence gene profile was observed, comprising: *cpa, emm, fbpt54, fctA, fctB, grab, hasA, hasB, hasC, ideS/mac, lepA, lmb, mf/spd, mf3, scpA, sda, sic, ska, slo, smeZ*, sp*eA*, sp*eB*, sp*eG*, sp*eJ*, and *srtC1*. This clade also exhibited the highest number of prophages and mobile genetic elements, indicating a potential increased genetic plasticity (**Figure 1**). Three prophages were recurrently detected in the emm1-like clades, including M1UK: T12 (carrying *speA*), P9, and 315.3 (carrying *spd* and *mf*). Similarly, emm3-, emm4- and emm12-like lineages showed enrichment in prophages and virulence genes.

The sp*eA* gene in emm1.0 strains did not show any mutations, unlike other strains that carried a single-nucleotide variant (A>G at position 984694) or multiple nucleotide changes (2406G0293, 2407G0310) (see **Supplementary Data 2**). Conversely, the *speJ* gene displayed sequence variation in all clades except emm1.0 and emm44.0, where it remained conserved.

Antimicrobial resistance (AMR) genes (highlighted in red in **Figure 1**) were detected in a minority of isolates and appeared to be sporadically associated with certain emm types. Macrolide resistance genes (*ermB*, *mefE*) were found in almost all isolates, occasionally alongside tetracycline resistance genes (*tetM*, *tetO*). Of note, emm1.0 and M1UK strains carried AMR genes, supporting the hypothesis that increased virulence, rather than antimicrobial resistance, drives their clinical relevance.

In summary, our data highlight a genetically diverse *S. pyogenes* population, with distinct clonal signatures and varying patterns of virulence, resistance, and genomic mobility.

### Dissection of Italian iGAS in pre- and post-pandemic period

A comprehensive overview of iGAS in Italy from 1994 to 2025 was depicted in **Table 1** which summarized all documented studies on invasive *S. pyogenes* molecularly characterized. One of the earliest comprehensive studies, conducted by Creti and colleagues (2007, Rome), examined 102 iGAS isolates collected over three intervals (1994–1996, 1997–2002, and 2003–2005), employing in-house PCR sequencing for emm typing (Creti et al., 2007). The isolates were obtained from sterile sites (blood, CSF, deep tissues), or from non-sterile sources in cases of toxic shock-like syndrome. Dominant emm types during this period included emm1, emm3, emm6, emm12, emm18, and emm89.

**Table 1:** Summary of the Italian investigation of invasive GAS.

Study	N. iGAS detected	N. sequenced iGAS isolates	Years of recruitment	Infection site	emm type detected	Molecular method	Bioproject number	Sequence availability
(Creti et al., 2007)	102	89 **^a^**	1994 to 1996; 1997 to 2002; 2003 to 2005	“A case of invasive GAS disease was defined as isolation of the bacterium from a site that is normally sterile, like blood, cerebrospinal fluid, joint aspirates, pericardial and peritoneal fluids, bone, deep tissues, or abscesses, at the time of surgery or necropsy. In case of toxic shock-like syndrome, GAS strains isolated from a nonsterile site (such as the skin, throat, or vagina) were also included.”	**^b^**1994-1996: emm1 (16), emm3 (6), emm4 (3), emm6 (5), emm12 (4), emm18 (1), emm89 (19) 1997-2002: emm1 (8), emm3 (7), emm6 (1), emm18 (2), emm89 (9) 2003-2005: emm1 (17), emm3 (9), emm4 (8), emm6 (5), emm12 (11), emm18 (7), emm89 (2)	PCR	NA	NA
(Mangioni et al., 2023)	19	19	2022 to 2023	blood cultures, intraoperative cultures	M1 global (3/19), emm28.0 (3/19), emm164.2 (3/19), M1UK (2/19), M1DK (1/19), emm4.0 (1/19), emm 11.0 (1/19), emm22.0 (1/19), emm58.0 (1/19), emm82.0 (1/19), emm87.0 (1/19), emm92.0 (1/19)	WGS	PRJEB63359	yes
(Vrenna et al., 2024)	19	6	2022 to 2024	blood (3), cerebrospinal fluid (1), pleural fluid (1), synovial liquid (1), ear (1), skin (1)	emm1 (5/6), emm75.0 (1/6)	WGS	–	no
(Arcari et al., 2025)	15	15	2023 to 2024	blood cultures (14), cerebrospinal fluid (1)	emm1.0 (6/15), emm12.0 (2/15), emm28.0 (2/14), emm6.4 (1/15), emm11.0 (1/15), emm75.0 (1/15), emm89.0 (1/15), emm94.1 (1/15)	WGS	PRJNA1070447	yes
(Bonomo et al., 2025)	35	35	2022 to 2024	Blood coltures (34), ear purulent swab (1)	emm 1 (13/35), emm 3 (2/35), emm 8 (1/35), emm 12 (3/35), emm 22 (1/35), emm 28 (3/35), emm 63 (1/35), emm 75 (2/35), emm 76 (1/35), emm 77 (1/35), emm 87 (2/35), emm 89 (2/35), emm 90 (2/35), emm 480 (1/35)	WGS	PRJNA193569	yes
(Corbella et al., 2025)	45	34	2015 to 2024	blood cultures	emm1.0 (11/34), emm12.0 (5/34), emm4.0 (4/34), emm89 (3/34), emm3.0 (2/34), emm2 (1/34), emm6 (1/34), emm9 (1/34), emm18 (1/34), emm22 (1/34), emm28 (1/34), emm44 (1/34), emm92 (1/34), emm118 (1/34)	WGS	PRJNA1170563 only assembly	no

**^a^**Isolates were screened by PCR.

**^b^**Most common emm types.

Summary of 6 invasive GAS studies conducted in Italy from 1994-2024. For each study, the following parameters are reported: total number of invasive GAS isolates detected, number of isolates subjected to WGS or molecular typing, recruitment period, anatomical sites of infection (blood cultures, cerebrospinal fluid, pleural fluid, synovial fluid, intraoperative cultures, ear, skin), EMM types identified with frequency distribution, molecular characterization method employed (PCR or WGS), BioProject accession numbers, and sequence data availability status.

With the advancement of whole-genome sequencing (WGS), more recent studies have provided in-depth molecular data. Mangioni et al. (2023, Milan) analyzed 19 iGAS isolates from 2022–2023, including M1 global, M1UK (UK lineage), and M1DK (Danish lineage) strains (Mangioni et al., 2023). Vrenna et al. (2024, Rome) reported data on a pediatric cohort from 2022–2024, identifying emm1 as the dominant type among the six sequenced strains (Vrenna et al., 2024). Arcari et al. (2025, Varese) sequenced 15 isolates from 2023–2024, again identifying emm1 as the most prevalent (Arcari et al., 2025). Bonomo et al. (2025, Bologna and Imola) characterized 35 isolates, with a predominance of emm1 and the presence of emm3, emm12, emm28, and rare variants such as emm480 (Bonomo et al., 2025). Corbella et al. (2025, Pavia) provided data from a broader surveillance period (2015–2024), sequencing 34 strains and confirming the persistence of emm1 (Corbella et al., 2025).

**Figure 3:**
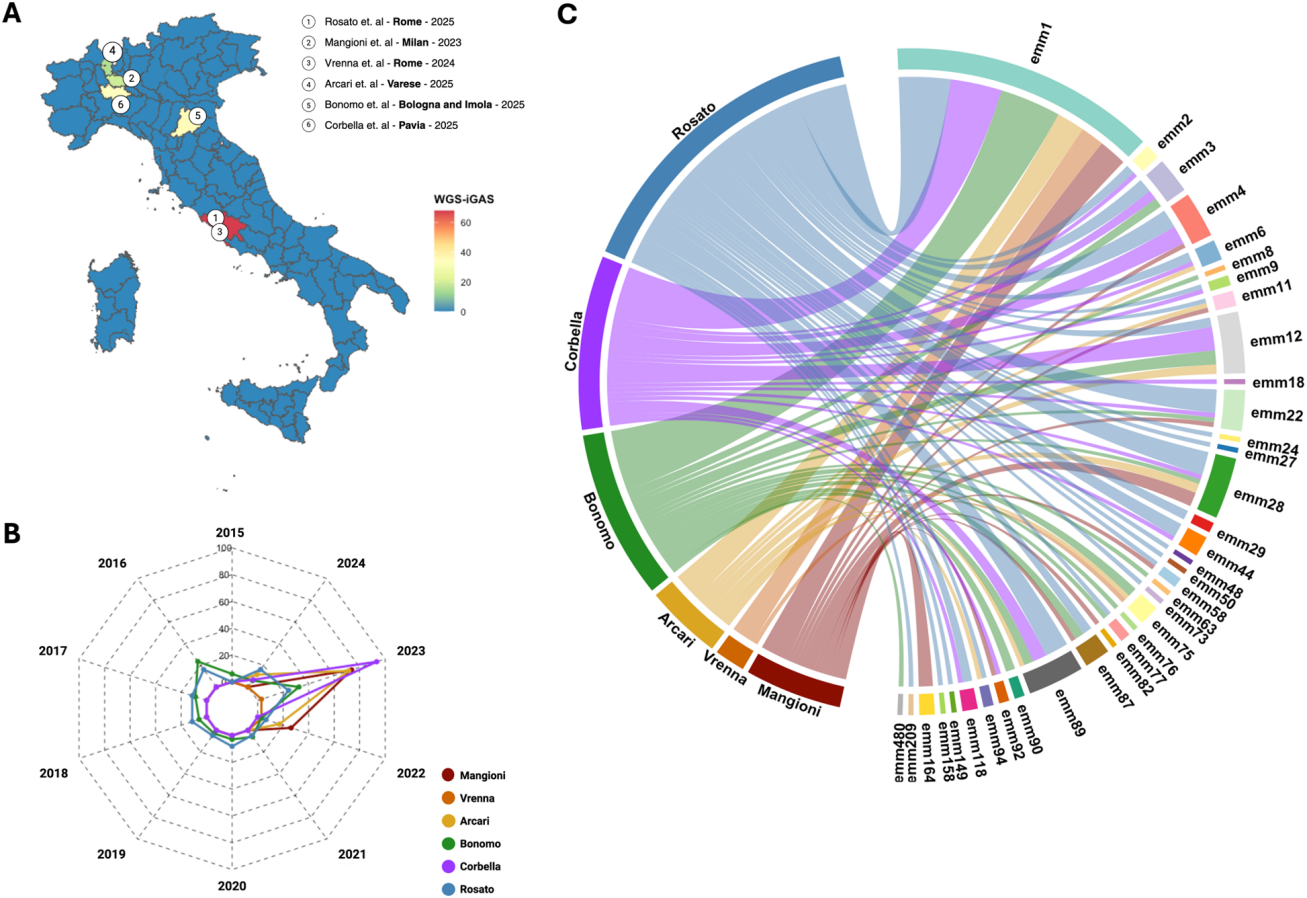
Geographic, temporal, and molecular distribution of molecularly characterized invasive GAS isolates in Italy. Data collected in **Table 1** showing whole genome sequenced invasive *Streptococcus pyogenes* (iGAS) were investigated to explore the Italian surveillance in pre- and post- pandemic period (2015 and 2024). Map of Italy showing the municipal distribution of iGAS isolates subjected to whole-genome sequencing (WGS). The color scale indicates the total number of sequenced isolates per municipal, ranging from blue (0 isolates) to red (>60 isolates) **(A)**. Spider chart illustrates the temporal distribution of WGS-iGAS isolates collected and sequenced by each center previously described in **Table 1** (Mangioni, Vrenna, Arcari, Bonomo, Corbella, Rosato – i.e. this study). The radial axis represents the percentage of sequenced isolates per year described by the authors **(B)**. Chord diagram showing the *emm* type distribution (right side) among the laboratories (left side). Ribbons represent the distribution and frequency of different *emm* types reported by each center, highlighting the molecular diversity of circulating iGAS strains **(C)**.

However, raw sequencing data were not consistently available across studies, and differences in sampling periods may limit direct comparisons. Despite this, a constant dominance of specific emm types, particularly emm1, was evident, while the broader genetic diversity of iGAS strains emphasized the importance of sustained molecular surveillance. Geographically, sequencing efforts have been uneven across Italy (**Figure 3A**). Most isolates originated from urban Rome, including the six pediatric cases from Vrenna and the 61 strains analyzed in the present study (2016–2024). Northern Italy remained underrepresented and Southern Italy absent, raising concerns about undetected iGAS incidence and gaps in genomic surveillance. The annual distribution of sequenced isolates (**Figure 3B**) showed low activity from 2015 to 2021 (1.19% to 10.12%), followed by a sharp increase in 2023 (48.21%) and a modest decline in 2024 (9.52%), which still exceeded pre-2022 levels. The chord diagram (**Figure 3C**) evidenced the diversity of circulating emm types across sequencing centers, with emm1, emm4, emm89, and emm12 being the most prevalent. While emm1 was ubiquitous, other types appeared regionally concentrated, supporting the existence of both widespread and localized iGAS lineages.

**Figure 4:**
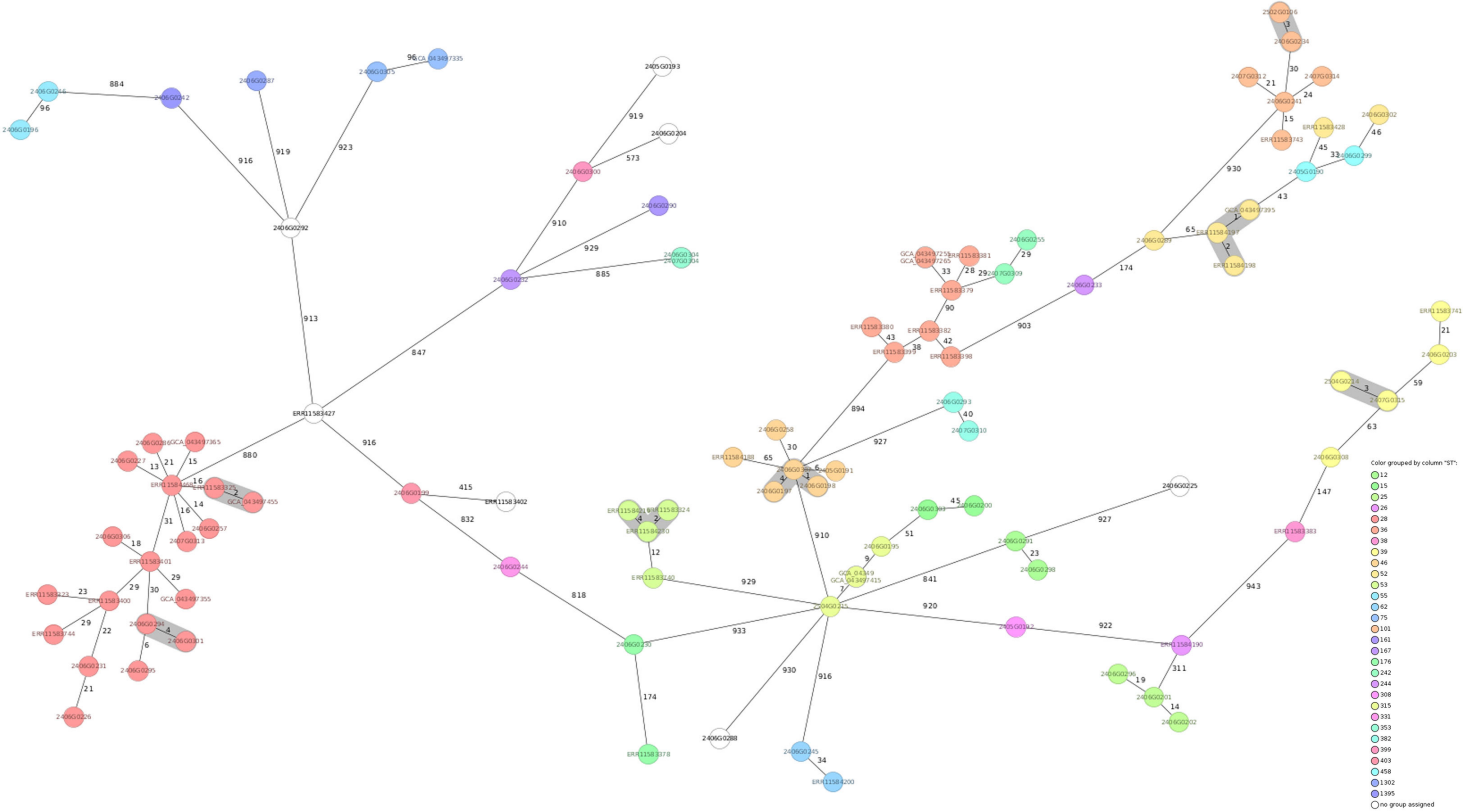
Phylogenetic relationship among the Italian molecularly characterized invasive GAS genomes. Minimum spanning tree generated from *de novo* assembled genomes of invasive *Streptococcus pyogenes* (iGAS) isolates, analyzed using core genome MLST (cgMLST) in Ridom SeqSphere+. Each node represents a single isolate, and the numbers on the edges indicate the number of allelic differences between isolates. Clusters of isolates differing by ≤6 single nucleotide variants (SNVs) were considered clonal and are gathered accordingly. Nodes were color-coded by sequence type (ST), as indicated in the legend.

To assess genetic relationships among strains, a core-genome multilocus sequence typing (cgMLST)–based minimum spanning tree was generated (**Figure 4**). Each node represents an isolate, color-coded by sequence type (ST), with edges labelled by SNP distances. The tree revealed several major clonal complexes (ST28, ST36, ST15, and ST39) characterized by short SNP distances, consistent with recent transmission or clonal expansion and slow evolution. Other sequence types, such as ST101 and ST452, appeared more genetically divergent or under sampled. Remarkably, genetically similar isolates were found in different geographical areas, suggesting interregional dissemination of dominant clones. ST15 and ST36 strains were found across distinct clusters but were separated by minimal SNP distances, further supporting recent spread. Central nodes connected various lineages, possibly representing ancestral strains involved in broader transmission chains.

Overall, the phylogenetic structure reflects both endemic persistence and dynamic evolution of iGAS in Italy, emphasizing the ongoing need for comprehensive genomic surveillance.

## Discussion

Despite numerous reports addressing the rise in GAS infections in the post-pandemic period, the underlying causes of this trend remain largely elusive. Several contributing factors appear to be more closely related to social rather than biological determinants: a marked decrease in Emergency Department visits across Europe between 2020 and 2022 (Golinelli et al., 2021; Paganini et al., 2022; Lopes et al., 2023; Schranz et al., 2023), an increased attention to respiratory infections in 2023 (De Maio et al., 2023), and the widespread adoption of non-pharmaceutical interventions during the COVID-19 pandemic events (Amuedo-Dorantes et al., 2021), interventions that have been co-associated with the changes in viral infections and the so-called “immunity debt” (Billard and Bont, 2023; Principi et al., 2023; E.C.f.D.P.a. Control, 2024).

Our previous data on a pediatric cohort aligned with the broader trend, highlighting an increase in GAS infections in 2023, with a sharp peak in cases during the winter and spring seasons and a shift toward preschool-aged children (Massese et al., 2024). Although there was an obvious increase, an extensive analysis suggested that this trend does not reflect a true general epidemiological surge (Johannesen et al., 2023). Indeed, examining the full 2016–2023 period in our center, the overall number of cases in 2023 was comparable to pre-pandemic years, with similar seasonal patterns (U.H.S. Agency, 2024).

However, systematic comparisons with pre-COVID data are still lacking, and investigations focusing on the etiological agent remain limited: for example we have noted no specific emm type association with the upsurge of GAS pediatric pharyngitis (De Maio et al., 2025b). Additionally, this data remains significantly impaired by national surveillance: in the UK the only peak was measured in the post pandemic period in comparison with pre pandemic or in 2024-2025 (U.H.S. UK Health Security Agency, 2025); conversely a replacement of the M1UK with emm3.93 strains has been observed (Davies et al., 2025).

Our comprehensive genomic and epidemiological characterization revealed a genetically diverse iGAS population with multiple emm types. Despite its recognized virulence, the M1UK lineage did not show a significant increase in the post-pandemic period. We provided a distinction of GAS infections in iGAS (accordingly to UKHSA guidelines), rGAS (Respiratory GAS infections) and oGAS (Other GAS infections, regarding infections from non-respiratory and non-sterile sites). On the contrary, Arcari et al. proposed a diverse classification based on GAS severity (Arcari et al., 2025), underscoring the significant influence of surveillance definitions on the perceived GAS epidemiology.

Although, we reported a predominance of emm1 among iGAS isolates, the considerable heterogeneity existing across the Italian studies (sampling periods, sequencing methodologies, and geographic coverage) does not support the comparison between the pre- and post-pandemic periods. While studies such as those by Vrenna et al. (Vrenna et al., 2024), Arcari et al (Arcari et al., 2025), Bonomo et al. (Bonomo et al., 2025), and Corbella et al. (2025) contribute valuable insights, most are based on short surveillance windows and small sample sizes. In contrast to the structured surveillance systems established in countries like the United Kingdom, France, and the Netherlands, Italy currently lacks a national iGAS reporting framework.

The observed clonal diversity supports the idea that iGAS is not driven by a single lineage, but rather by a mosaic of evolving clones with varied genetic compositions. Importantly, AMR genes were limited and largely confined to both emm1.0 and non-emm1.0 lineages, supporting the idea that virulence shapes the clinical impact of iGAS more than antimicrobial resistance. This finding is particularly significant considering recent global concerns regarding rising antibiotic resistance, even though GAS resistance to macrolide has been included as a WHO priority pathogen (Salam et al., 2023; G.B.D.L.R. Infections and C. Antimicrobial Resistance, 2024).

Our findings underscore the urgent need for a national genomic surveillance program that integrates clinical, microbiological, and genomic data. Indeed, M1UK strains were already detected in the UK around 2010, and the incidence was observed to increase in the past years and after the pandemic event (Vieira et al., 2024). Intriguingly, the rapid expansion of M1UK may depend on the high bacterial fitness due to less frequent regulatory mutations, rather than to the acquisition of virulence genes (i.e. *spd1* or *speC*) (Turner et al., 2017; Vieira et al., 2024). The higher production of the SpeA toxin contribute to scarlet fever increased incidence (Turner et al., 2017; Lynskey et al., 2019). Nevertheless, our results are opposite to Walker et al. (2014) that observed a DNase encoding gene (*sda1*) providing a selective pressure for *covR/S* mutations favoring a switch to iGAS infection. In other words, GAS pathogenicity is not directly dependent on the presence of virulence genes, but rather on the regulatory systems that control their expression. For instance, intermediate levels of virulence can maximize bacterial transmission while avoiding host clearance (Wollein Waldetoft and Raberg, 2014). Furthermore, transcriptional regulators coordinate virulence gene expression according to the host environment, allowing the transition between colonization and disease conditions (Kreikemeyer et al., 2003; Kachroo et al., 2019).

Mutations in virulence-associated regulatory genes are highly prevalent among *S. pyogenes* isolates, suggesting a fine-tuning of gene function, rather than complete gain or loss of function. In other words, the high mutation frequency in *covS* may be consistent with the enhanced expression of numerous virulence factors. This may be counterbalanced by *covR* mutational frequency potentially reflecting evolutionary constrains to preserve its core repressor function (Dalton and Scott, 2004; Trevino et al., 2009; Bao et al., 2015; Horstmann et al., 2022).

Mutations in *rexA* and *rexB* may promote advantages during infection, particularly in environments with high phagocytic pressure; as well as variations in *rgg* and *srv* may be support stress adaptations (Bitoun et al., 2011; Zhu et al., 2018; Franza et al., 2021). Interestingly, except for *rexB* gene, variations in these genes appear to balance mutations in *covS*.

Additionally, the total mutational landscape highlighted key evolutionary dynamics shaping the genome of *S. pyogenes*. The broad distribution of SNVs and MNVs, particularly their presence in high frequency bins, suggests that subtle sequence changes are tolerated or even beneficial in certain genomic contexts. These variants may contribute to phenotypic diversity and host adaptation without compromising essential functions. In contrast, the low prevalence of insertions, deletions, and replacements at higher frequencies implies that more disruptive structural mutations may reflect either recent occurrence or strong purifying selection acting against them due to deleterious effects on gene integrity, particularly in essential regulatory regions. The differential frequency distributions across mutation types underscore the balance between genetic diversification (SNVs/MNVs) and functional constraint (indels). Adaptive changes may preferentially arise through point mutations in regulatory genes while maintaining overall genomic stability and may be considered as early genomic warnings for hypervirulent strains. Unfortunately, how they impact on the pathogenetic processes remains elusive. If wide accessory genome may explain a different virulence pattern, the strong relationship between *S. pyogenes* and other bacteria, and possible transfer of genetical material may significantly impair its genomic evolution and pathogenicity (Jackson et al., 2011; Segerman, 2012).

In this context, the lowest number of mutations in emm type 1-like, including M1UK strains, may confer a selective advantage in the host-pathogen interactions, corroborating the idea that M1UK clade success may be related to low genetic plasticity in comparison with other lineages at least in regulatory operons.

Our data, together with findings from other Italian studies, capture the genetic and epidemiological picture of *S. pyogenes* circulating in Italy, placing it within the broader European context defined by recent large-scale studies. Despite uneven sampling across years, the absence of detailed clinical metadata still limits our ability to link genomic features with patient-level outcomes. Establishing multicenter genomic collaborations will be essential to broaden the scope and impact of iGAS surveillance. Future research should further elucidate the functional consequences of regulatory gene mutations and determine how these alterations influence bacterial fitness and virulence.

## Materials and methods

### Sample enrollment

*S. pyogenes* strains isolated from blood and cerebrospinal fluid in the period 2016–2024 at the Microbiology institute of the Fondazione Policlinico A. Gemelli IRCCS were genotypically characterized. iGAS cases were defined accordingly to UK guidelines (U.H.S. UK Health Security Agency, 2023), which requires the detection of *S. pyogenes* from a normally sterile site such as blood, cerebrospinal fluid, joint aspirate, pericardial, peritoneal or pleural fluids, bone, deep tissue, or deep abscess. No *S. pyogenes* strain was isolated from other sterile fluids. Demographic (sex and age) and hospital data (ward) were collected for all the strains included in the study (**Supplementary Table 1**). Each bacterial isolate was identified by using Matrix-Assisted Laser Desorption/Ionization Time-of-Flight Mass Spectrometry (MALDI-ToF MS, BrukerDaltonics) following first culture detection, as above-described (Mannavola et al., 2025). Clinical strains were finally collected in cryotubes containing 20% glycerol and stored at -80 °C for further analyses.

### Invasive GAS specimens’ manipulation, genomic DNA extraction and whole genome sequencing

To perform whole genome sequencing (WGS), frozen strains were streaked onto Columbia blood agar (Oxoid) prior to prepare broth culture by using Todd-Hewitt broth (Oxoid) liquid medium. Sixty-one strains out of sixty-three were recovered and genomic DNA was extracted from cultures grown overnight by using DANAGENE Microbial DNA kit (Danagen-Bioted) according to manufacturer’s instructions. Briefly, 10 ml of overnight cultures were centrifuged; the bacterial pellet was harvested and re-suspended in CTAB lysis buffer. All procedures were performed in a biosafety level 2 laboratory. DNA concentration and purity were assessed with a NanoDrop One spectrophotometer (ThermoFisher) before proceeding with the library preparation, that was carried out by using the Nextera DNA Prep kit (Illumina), as described above (Posteraro et al., 2024; De Maio et al., 2025a). 250 bp paired end reads were generated by an Illumina MiSeq DX platform (De Maio et al., 2025a). Fastq sequences have been deposited in the NCBI Sequence Read Archive (BioProject accession number: PRJNA1294401).

### Whole genome sequencing bioinformatic analysis

Raw data were processed using CLC Genomic Workbench (Qiagen). Each sample was checked following a quality step (quality limit: 0.05, maximum number of ambiguities: 2) and Illumina adapter trimming process. Quality checked trimmed reads followed a k-mer analysis to exclude possible contaminations, and best matched reads with *S. pyogenes* genomes were collected for the next steps.

The high-quality reads resulted from this pre-processing were used for *de novo* genome assembly (bubble size:50, word size: 20, minimum contig length 200). To control the assembly process, for each sample pre-processed reads were mapped to the obtained contigs. *De novo* assemblies were used to detected virulence genes and antimicrobial resistance genes by ABRicate v.1.0.1 (https://github.com/tseemann/abricate). The latter software was used with Comprehensive Antibiotic Resistance Database (CARD, 2631 sequences updated to 2024-Jul-17) (Jia et al., 2017) and Virulence Factor Database (VFDB, 2597 sequences updated to 2024-Jul-18) (Chen et al., 2016). Short-read assemblies were finally analyzed to determine the ST using a 1095-loci multilocus sequence typing (MLST) scheme (Toorop et al., 2023), as implemented in Ridom SeqSphere+ (*ridoma bionformatics*) (Kohl et al., 2014). A minimum spanning tree, based on the core-genome MLST profiles, was generated to visualize phylogenetic distance between clinical strains. Emm-typer was used to detect EMM-Cluster and EMM-Type for each strain (https://github.com/MDU-PHL/emmtyper). Mobilome analysis was carried out as follows: the presence of integrative and conjugative elements (ICEs) and of integrative and mobilizable elements (IMEs) in the assemblies was investigated with Mobile Genetic Element finder (MGEFinder) (Durrant et al., 2020), while the presence of prophages was investigated with PhySpy (Akhter et al., 2012). M1UK Clone was finally detected mapping each strain sequence with *Streptococcus pyogenes* MGAS5005 (Accession number CP000017.2) reference genome according to Li et al. (Li et al., 2023). A neighbor-joining tree was generated to summarize phylogenetic relationships and annotate metadata using *ggtree* package v.3.8.2. Default parameters were used for all software unless otherwise specified. The distribution and frequency of mutation types in *S. pyogenes emm1* genomes in comparison with the reference strain was detected. Different mutation types were identified: single nucleotide variant (SNV), multi-nucleotide variant (MNV), insertions, deletions, and replacement events; while their frequency accounted as: <5%, 5–20%, 20–50%, 50–80%, and >80%. All software and packages used for the genomic analysis were reported in **Supplementary Table 2**.

## Data Availability

The datasets presented in this study can be found in online repositories. The names of the repository/repositories and accession number(s) can be found in the article/**Supplementary Material**.

## Supplementary material

Supplementary Table 1 Epidemiological features of the invasive GAS isolates subjected to Whole Genome Sequencing in our center. Clinical and molecular characteristics of invasive GAS isolates collected in Italy from 2016-2024. Patient demographics (age, gender), source of GAS clinical isolation, hospital ward of origin, sequence type (ST), EMM type, and EMM cluster classification are reported for each isolate. SCVC, short-term central venous catheter; SCVP, short-term central venous port; PICC, peripherally inserted central catheter.

Supplementary Table 2 List of the software and packages used for the GAS isolates genomic characterization.

Supplementary Figure 1 Heatmap representing the distribution and frequency of mutation types in *Streptococcus pyogenes* emm1 genomes in comparison with the reference strain. Each row corresponds to a different mutation type, including single nucleotide variant (SNV), multi-nucleotide variant (MNV), insertions, deletions, and replacement events. Columns represent bins of increasing frequency among the sequenced isolates: <5%, 5–20%, 20–50%, 50–80%, and >80%. The color scale, ranging from yellow (high counts) to dark red (low counts), indicates the absolute number of mutations observed within each frequency bin.

Supplementary Data Sheet 1 List of the invasive GAS isolates sequenced in this study or retrieved by NCBI database.

Supplementary Data Sheet 2 Dataset of genetic variants found in invasive emm1 GAS isolates sequenced in this study in comparison with reference strain.

Supplementary Data Sheet 3 Overview of mutations identified in virulence regulatory genes of invasive GAS isolates sequenced in this study in comparison with reference strain.
