# Supplementary material for: The invasive GAS puzzle in Italy: genomic insights from a hospital cohort in a fragmented surveillance landscape
Source: Front Cell Infect Microbiol. 2026 Jan 7;15:1684665. doi: 10.3389/fcimb.2025.1684665 (PMC12819700; doi:10.3389/fcimb.2025.1684665)
Supplement: Supplementary Table 1 — Epidemiological features of the invasive GAS isolates subjected to Whole Genome Sequencing in our center. Clinical and molecular characteristics of invasive GAS isolates collected in Italy from 2016-2024. Patient demographics (age, gender), source of GAS clinical isolation, hospital ward of origin, sequence type (ST), EMM type, and EMM cluster classification are reported for each isolate. SCVC, short-term central venous catheter; SCVP, short-term central venous port; PICC, peripherally inserted central catheter. [file Table1.docx]

| **Study** | **N. iGAS detected** | **N. sequenced iGAS isolates** | **Years of recruitment** | **Infection Site** | **emm type  detected** | **Molecular method** | **Bioproject Number** | **Sequence Availability** |
| --- | --- | --- | --- | --- | --- | --- | --- | --- |
| [Creti 2007] | 102 | 89 **^a^** | 1994 to 1996; 1997 to 2002; 2003 to 2005 | "A case of invasive GAS disease was defined as isolation of the bacterium from a site that is normally sterile, like blood, cerebrospinal fluid, joint aspirates, pericardial and peritoneal fluids, bone, deep tissues, or abscesses, at the time of surgery or necropsy. In case of toxic shock-like syndrome, GAS strains isolated from a nonsterile site (such as the skin, throat, or vagina) were also included." | **^b^**1994-1996: emm1 (16), emm3 (6), emm4 (3), emm6 (5), emm12 (4), emm18 (1), emm89 (19) 1997-2002: emm1 (8), emm3 (7), emm6 (1), emm18 (2), emm89 (9) 2003-2005: emm1 (17), emm3 (9), emm4 (8), emm6 (5), emm12 (11), emm18 (7), emm89 (2) | PCR | NA | NA |
| [Mangioni 2023] | 19 | 19 | 2022 to 2023 | blood cultures, intraoperative cultures | M1 global (3/19), emm28.0 (3/19), emm164.2 (3/19), M1UK (2/19), M1DK (1/19), emm4.0 (1/19), emm 11.0 (1/19), emm22.0 (1/19), emm58.0 (1/19), emm82.0 (1/19), emm87.0 (1/19), emm92.0 (1/19) | WGS | PRJEB63359 | yes |
| [Vrenna 2024] | 19 | 6 | 2022 to 2024 | blood (3), cerebrospinal fluid (1), pleural fluid (1), synovial liquid (1), ear (1), skin (1) | emm1 (5/6), emm75.0 (1/6) | WGS | - | no |
| [Arcari 2025] | 15 | 15 | 2023 to 2024 | blood cultures (14), cerebrospinal fluid (1) | emm1.0 (6/15), emm12.0 (2/15), emm28.0 (2/14), emm6.4 (1/15), emm11.0 (1/15), emm75.0 (1/15), emm89.0 (1/15), emm94.1 (1/15) | WGS | PRJNA1070447 | yes |
| [Bonomo 2025] | 35 | 35 | 2022 to 2024 | Blood coltures (34), ear purulent swab (1) | emm 1 (13/35), emm 3 (2/35), emm 8 (1/35), emm 12 (3/35), emm 22 (1/35), emm 28 (3/35), emm 63 (1/35), emm 75 (2/35), emm 76 (1/35), emm 77 (1/35), emm 87 (2/35), emm 89 (2/35), emm 90 (2/35), emm 480 (1/35) | WGS | PRJNA193569 | yes |
| [Corbella 2025] | 45 | 34 | 2015 to 2024 | blood cultures | emm1.0 (11/34), emm12.0 (5/34), emm4.0 (4/34), emm89 (3/34), emm3.0 (2/34), emm2 (1/34), emm6 (1/34), emm9 (1/34), emm18 (1/34), emm22 (1/34), emm28 (1/34), emm44 (1/34), emm92 (1/34), emm118 (1/34) | WGS | PRJNA1170563  only assembly | no |

**^a^**isolates were screened by PCR

**^b^**most common emm types
