# Supplementary material for: The invasive GAS puzzle in Italy: genomic insights from a hospital cohort in a fragmented surveillance landscape
Source: Front Cell Infect Microbiol. 2026 Jan 7;15:1684665. doi: 10.3389/fcimb.2025.1684665 (PMC12819700; doi:10.3389/fcimb.2025.1684665)
Supplement: Supplementary Table 2 — List of the software and packages used for the GAS isolates genomic characterization. [file Table2.docx]

| **Year** | **Code** | **Age** | **Gender** | **Source** | **Ward** | **ST** | **EMM type** | **EMM_cluster** |
| --- | --- | --- | --- | --- | --- | --- | --- | --- |
| **2016** | 2406G0289 | 53 | F | SCVC | Emergency Department | ST52 | EMM28.0 | E4 |
|  | 2406G0291 | 78 | F | blood | Orthopedics And Traumatology | ST12 | EMM29.8 | Y |
|  | 2406G0293 | 62 | F | blood | Emergency Department | ST382 | EMM6.4 | Y |
|  | 2406G0294 | 69 | M | SCVC | Emergency Department | ST28 | EMM1.18 | A-C3 |
|  | 2406G0296 | 79 | M | blood | Emergency Department | ST25 | EMM44.0 | E3 |
|  | 2406G0298 | 82 | F | blood | Emergency Department | ST12 | EMM29.8 | Y |
|  | 2406G0302 | 39 | F | blood | Obstetrics | ST52 | EMM28.0 | E4 |
|  | 2406G0303 | 83 | M | blood | Emergency Department | ST15 | EMM3.1 | A-C5 |
|  | 2406G0304 | 52 | F | blood | Emergency Department | ST353 | EMM209.0 | E3 |
| **2017** | 2406G0288 | 53 | M | SCVC | Hematology | ST70 | EMM24.4 | Y |
|  | 2406G0292 | 56 | F | SCVC | Day Hospital Radiation And Chemotherapy | ST599 | EMM87.9 | E3 |
|  | 2406G0295 | 5 | F | SCVC | Pediatric Emergency And Trauma Center | ST28 | EMM1.18 | A-C3 |
|  | 2406G0299 | 81 | F | blood | Geriatrics | ST458 | EMM28.0 | E4 |
|  | 2406G0300 | 57 | M | blood | Emergency Department | ST399 | EMM77.0 | E4 |
|  | 2406G0305 | 57 | F | blood | Nephrology – Columbus Unit | ST75 | EMM9.0 | E3 |
|  | 2406G0306 | 66 | M | blood | Emergency Department | ST28 | EMM1.0 | A-C3 |
| **2018** | 2406G0286 | 80 | M | blood | Emergency Department | ST28 | EMM1.24 – M1UK^12SNPs^ | A-C3 |
|  | 2406G0287 | 51 | M | blood | Internal Medicine And Gastroenterology | ST1302 | EMM158.0 | E6 |
|  | 2406G0290 | 64 | M | blood | Emergency Department | ST161 | EMM48.1 | E6 |
|  | 2406G0301 | 48 | M | blood | Emergency Department | ST28 | EMM1.18 | A-C3 |
|  | 2406G0307 | 78 | F | blood | Emergency Department | ST46 | EMM22.21 | E4 |
|  | 2406G0308 | 66 | M | blood | Emergency Department | ST39 | EMM4.0 | E1 |
| **2019** | 2406G0196 | 51 | M | blood | General Medicine | ST55 | EMM2.0 | E4 |
|  | 2406G0202 | 57 | F | blood | Day Hospital Gynecologic Oncology | ST25 | EMM44.0 | E3 |
|  | 2406G0203 | 55 | F | blood | Postoperative Intensive Care Unit | ST39 | EMM4.19 | E1 |
| **2020** | 2406G0195 | 52 | M | blood | Emergency Department | ST315 | EMM3.93 | A-C5 |
|  | 2406G0197 | 76 | F | blood | Emergency Department | ST46 | EMM22.21 | E4 |
|  | 2406G0198 | 72 | F | blood | Emergency Department | ST46 | EMM22.21 | E4 |
|  | 2406G0201 | 89 | M | blood | Emergency Department | ST25 | EMM44.0 | E3 |
|  | 2406G0258 | 71 | F | blood | Emergency Department | ST46 | EMM22.0 | E4 |
| **2021** | 2406G0199 | 77 | F | SCVP | Emergency Department | ST403 | EMM11.1 | E6 |
|  | 2406G0200 | 59 | M | blood | Emergency Department | ST15 | EMM3.1 | A-C5 |
|  | 2406G0204 | 71 | M | SCVP | Neurosurgery | ST714 | EMM149.0 | none |
| **2022** | 2405G0190 | 40 | F | SCVP | Emergency Department | ST458 | EMM28.0 | E4 |
|  | 2405G0191 | 65 | F | SCVP | Emergency Department | ST46 | EMM22.21 | E4 |
|  | 2405G0192 | 35 | F | SCVP | Emergency Department | ST308 | EMM27.0 | E2 |
|  | 2405G0193 | 64 | M | SCVP | Emergency Department | ST565 | EMM118.2 | E3 |
| **2023** | 2406G0225 | 73 | F | SCVP2 | Emergency Department | ST89 | EMM94.1 | E6 |
|  | 2406G0226 | 1 | M | SCVP | Emergency Department | ST28 | EMM1.25 | A-C3 |
|  | 2406G0227 | 68 | M | SCVP | Emergency Department | ST28 | EMM1.0 – M1UK^26SNPs^ | A-C3 |
|  | 2406G0230 | 69 | M | SCVP | Cardiology – Subintensive Care | ST176 | EMM58.0 | E3 |
|  | 2406G0231 | 65 | F | SCVP | Emergency Department | ST28 | EMM1.25 | A-C3 |
|  | 2406G0232 | 60 | F | SCVP | Emergency Department | ST167 | EMM118.0 | E3 |
|  | 2406G0233 | 77 | M | PICC | Gastroenterology | ST244 | EMM28.0 | E4 |
|  | 2406G0234 | 73 | M | SCVP | Geriatrics | ST101 | EMM89.0 | E4 |
|  | 2406G0241 | 64 | F | SCVP2 | Internal Medicine – Geriatrics | ST101 | EMM89.0 | E4 |
|  | 2406G0242 | 45 | M | SCVP | Neurology | ST1395 | EMM50.0 | E2 |
|  | 2406G0244 | 59 | M | SCVP | Emergency Department | ST331 | EMM73.0 | E4 |
|  | 2406G0245 | 86 | F | SCVP | Emergency Department | ST62 | EMM87.0 | E3 |
|  | 2406G0246 | 73 | M | SCVP | Orthopedics | ST55 | EMM2.0 | E4 |
|  | 2406G0255 | 85 | F | SCVP | Emergency Department | ST242 | EMM12.37 | A-C4 |
|  | 2406G0257 | 50 | M | SCVP | Emergency Department | ST28 | EMM1.0 – M1UK^26SNPs^ | A-C3 |
| **2024** | 2407G0309 | 70 | M | SCVP | Emergency Department | ST242 | EMM12.101 | A-C4 |
|  | 2407G0310 | 32 | F | SCVP | Obstetric Pathology | ST382 | EMM6.0 | Y |
|  | 2407G0311 | 56 | M | SCVP | Emergency Department | ST28 | EMM1.0 – M1UK^26SNPs^ | A-C3 |
|  | 2407G0312 | 60 | F | PICC | Neurology | ST101 | EMM89.0 | E4 |
|  | 2407G0313 | 31 | F | SCVP | Obstetric And Gynecologic Emergency Department | ST28 | EMM1.0 – M1UK^26SNPs^ | A-C3 |
|  | 2407G0314 | 88 | M | SCVP | Emergency Department | ST101 | EMM89.0 | E4 |
|  | 2407G0315 | 67 | M | SCVP | Emergency Department | ST39 | EMM4.0 | E1 |
|  | 2502G0106 | 63 | F | SCVP | Emergency Department | ST101 | EMM89.0 | E4 |
|  | 2504G0214 | 66 | F | SCVP | Emergency Department | ST39 | EMM4.0 | E1 |
