# Supplementary material for: The invasive GAS puzzle in Italy: genomic insights from a hospital cohort in a fragmented surveillance landscape
Source: Front Cell Infect Microbiol. 2026 Jan 7;15:1684665. doi: 10.3389/fcimb.2025.1684665 (PMC12819700; doi:10.3389/fcimb.2025.1684665)
Supplement: Supplementary file 3 [file Table3.docx]

| **Whole genome sequencing** | | |
| --- | --- | --- |
| **Package** | **Reference** | **WebSite** |
| CLC Genomic Workbench | Bioinformatics software | https://digitalinsights.qiagen.com/products-overview/discovery-insights-portfolio/qiagen-clc-genomics |
| Ridom SeqSphere+ | Bioinformatics software | Ridom SeqSphere+ - Overview |
| ABRicate | Mass screening of contigs for antimicrobial resistance or virulence genes | https://github.com/tseemann/abricate |
|  |  |  |
| Comprehensive Antibiotic Resistance Database (CARD) | 10.1093/nar/gkw1004 | - |
| Virulence Factor Database (VFDB) | 10.1093/nar/gkv1239 | - |
| emmtyper | Command line tool for emm-typing of Streptococcus pyogenes using a de novo or complete assembly | https://github.com/MDU-PHL/emmtyper |
| Physpy | 10.1093/nar/gks406  10.5281/zenodo.3475717 | https://github.com/linsalrob/PhiSpy |
| MGEfinder | 10.1016/j.chom.2019.10.022 | https://github.com/bhattlab/MGEfinder |
