# Supplementary figures and images for: The invasive GAS puzzle in Italy: genomic insights from a hospital cohort in a fragmented surveillance landscape
Source: Front Cell Infect Microbiol. 2026 Jan 7;15:1684665. doi: 10.3389/fcimb.2025.1684665 (PMC12819700; doi:10.3389/fcimb.2025.1684665)

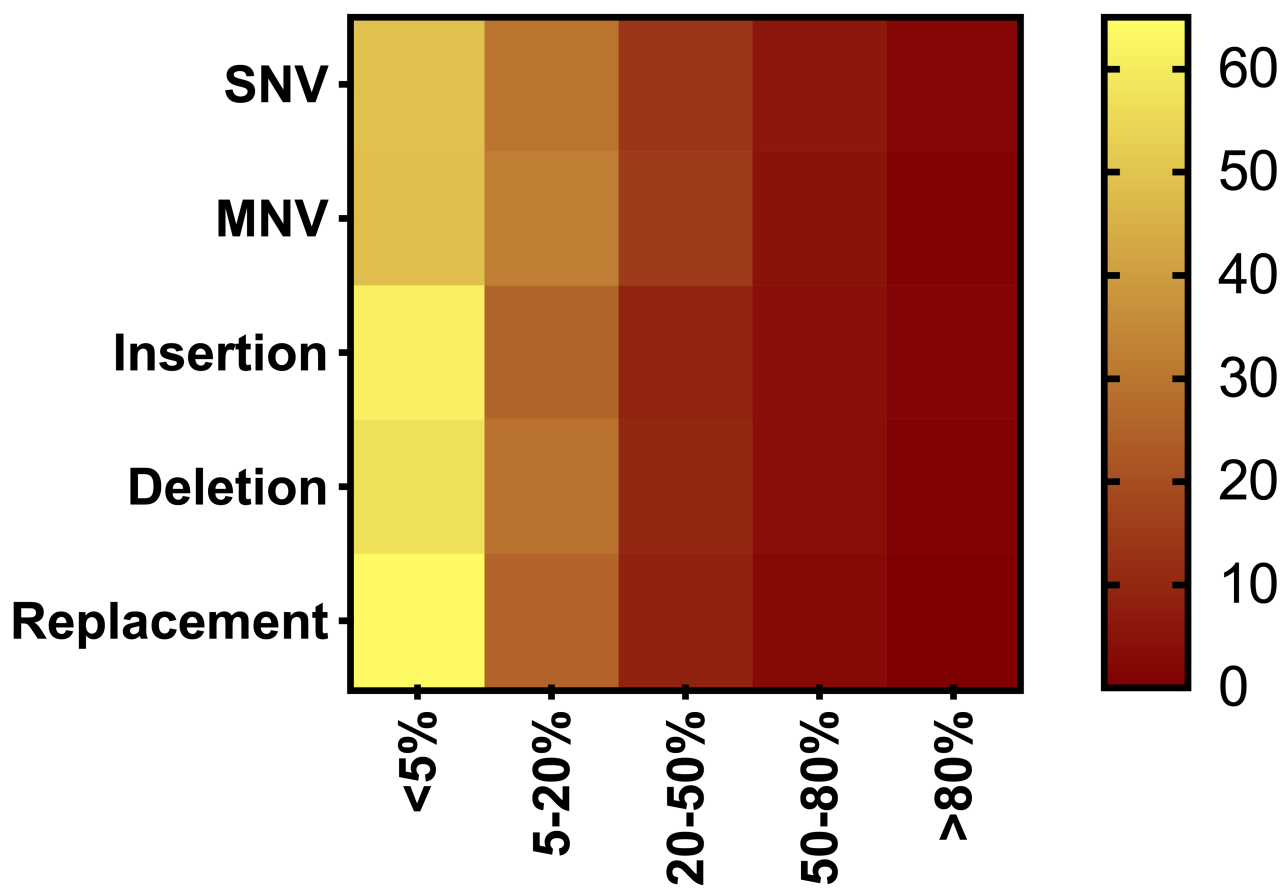

Supplement: Supplementary Figure 1 — Heatmap representing the distribution and frequency of mutation types in Streptococcus pyogenes emm1 genomes in comparison with the reference strain. Each row corresponds to a different mutation type, including single nucleotide variant (SNV), multi-nucleotide variant (MNV), insertions, deletions, and replacement events. Columns represent bins of increasing frequency among the sequenced isolates: <5%, 5–20%, 20–50%, 50–80%, and >80%. The color scale, ranging from yellow (high counts) to dark red (low counts), indicates the absolute number of mutations observed within each frequency bin. [file DataSheet1.pdf]
